# Operationalizing rapid implementation science for responsive HIV programs

**DOI:** 10.3389/frhs.2026.1860521

**Published:** 2026-07-09

**Authors:** Andrew Marvin Kanyike, Michael J. A. Reid, Rachel Sturke, Susan Vorkoper, Elvin H. Geng, Nyanyiwe Masingi Mbeye

**Affiliations:** 1 HIV, Infectious Disease and Global Health Implementation Research Institute, Washington University in St. Louis, St. Louis, MO, United States; 2 Department of Clinical and Biomedical Sciences, University of Exeter, Exeter, United Kingdom; 3 Bureau of Global Health Security and Diplomacy, President’s Emergency Plan for AIDS Relief, Washington, DC, United States; 4 Institute of Global Health Sciences, University of California at San Francisco, San Francisco, CA, United States; 5 Fogarty International Center, NIH, Bethesda, MD, United States; 6 Division of Infectious Diseases, Washington University in St Louis, St. Louis, MO, United States; 7 Evidence Informed Decision-making Centre, School of Global and Public Health, Kamuzu University of Health Sciences, Blantyre, Malawi

**Keywords:** evidence-based decision-making, HIV programmes, program-embedded research, rapid implementation science, timely evidence generation

## Abstract

**Introduction:**

HIV and other public health programs operate within dynamic, resource-constrained environments, requiring timely, evidence-informed decisions that align with service delivery. Rapid Implementation Science (RIS) provides a pragmatic approach to generating relevant, actionable evidence aligned with program needs. In this article, we propose a suite of rapid, fit-for-purpose methods and systems-level enablers to facilitate RIS. We synthesized examples of rapid research in HIV and global health that produced actionable findings.

**Main body:**

We propose a cascade for operationalising RIS within HIV and other public health programs. First, program-embedded formulation of research questions and regulatory governance, co-led by implementers, researchers, and decision-makers, to align with program priorities and timelines. Second, fit-for-purpose study designs, spanning rapid qualitative methods, mixed-methods frameworks, and pragmatic quantitative approaches, with iterative testing, early assessment of proximal outcomes, and adaptive learning without compromising rigour. Third, pre-prepared data systems, combined with fast, low-burden data collection techniques, enable timely analysis by leveraging routine clinical data, real-time dashboards, and mobile or crowdsourced tools. Finally, these approaches should generate decision-ready results that inform mid-course program adaptation, resource reallocation, and policy-relevant action.

**Conclusion:**

RIS should be leveraged to align scientific inquiry with the pace of real-world public health programs through building collaborative platforms, analysis-ready data infrastructure, and adopting innovative rapid study designs that can generate timely, rigorous, and decision-relevant evidence. Future efforts should expand testing of hybrid rapid methods, strengthen locally led capacity, and document trade-offs between speed and rigour.

## Introduction

The ability to rapidly generate new scientific knowledge, beyond simply accelerating the translation of existing evidence into practice, has become increasingly vital in public health and implementation science (1). This need is especially pronounced in contexts such as HIV programs, where service delivery systems are evolving rapidly (1). The introduction of new interventions often creates novel implementation challenges. For example, although oral pre-exposure prophylaxis (PrEP) has been extensively studied for over 15 years, the introduction of long-acting injectable formulations raises new operational questions, including how clinical workflows must be adapted and which patient groups may require targeted support to maximize its effectiveness (2, 3).

As new interventions are introduced at an increasingly rapid pace, implementation environments are simultaneously reshaped by shifting health systems, economic conditions, funding priorities, and evolving epidemic dynamics (4). Policy shifts also have narrow windows of opportunity in which timely evidence generation is essential. Current and shifting funding limitations exacerbate this urgency: constrained budgets, shorter grant cycles, and competing priorities mean that programs cannot afford lengthy delays in generating actionable evidence (5). When evidence arrives late, resources may already have been committed, interventions scaled, or funding windows closed, leaving programs without the data needed to guide adaptation. Consequently, evidence must be not only rigorous but also timely, relevant, and responsive to program needs.

In practice, however, implementation research remains largely misaligned with program timelines (4). It is often “out of our hands,” delayed by funding cycles, regulatory approvals, and data access constraints, so that evidence arrives after key implementation decisions have already been made. In other cases, research is “out of the loop,” asking technically sound questions but conducted outside program settings and timelines, limiting its capacity to inform real-time adaptation. Furthermore, highly rigorous trials, while scientifically robust, are frequently “overcooked,” producing definitive results only after programs have already scaled interventions nationally, thereby constraining their relevance. As a result, scientifically sound implementation science studies may still fail to meaningfully inform implementation, rendering them untimely, unresponsive, or of limited programmatic value.

This tension raises a central question for implementation science: can scientific research be both timely and rigorous? Rapid Implementation Science (RIS) offers a potentially critical approach for addressing the growing mismatch between the pace of scientific inquiry and the realities of programmatic decision-making (6). Smith et al. defined rapid implementation as the efficient and timely delivery of evidence-based programs or interventions by rethinking rigour and strategically adapting methods, procedures, implementation frameworks, and trial designs to align with specific research aims and objectives (6). RIS is guided by the 5Rs framework—Rapidity, Relevance, Responsiveness, Rigour, and Replicability—which articulates how implementation research can remain methodologically robust while aligning with real-world decision cycles (7).

However, the operationalization of RIS depends on the availability of appropriate methods and mechanisms for rapid study design and execution. We argue that through deliberate rethinking of how research questions are formulated, study designs are selected, analyses are conducted, and results are delivered within programs, it is possible to generate evidence that meets the 5Rs without compromising quality or transparency. The contribution of RIS does not lie in introducing new study designs or analytic techniques, but in articulating an integrated operational approach that aligns research governance, methodological choices, data systems, and dissemination processes with the decision-making timelines of real-world programs.

To advance this argument, we conducted a literature review, complemented by internal discussions during implementation science workshops and expert consultations, to examine the mechanisms underlying RIS, the methodologies that support it, and the contextual and structural enablers that can facilitate it. Based on this synthesis, we propose a four-step cascade as a practical framework for operationalising RIS within programs. This framework is intended for researchers, implementers, policymakers, and funders engaged in HIV and public health programs, and is particularly relevant for settings where implementation decisions must be made within constrained timelines, limited resources, and evolving policy environments.

### Rapid implementation science cascade

**Figure 1:**
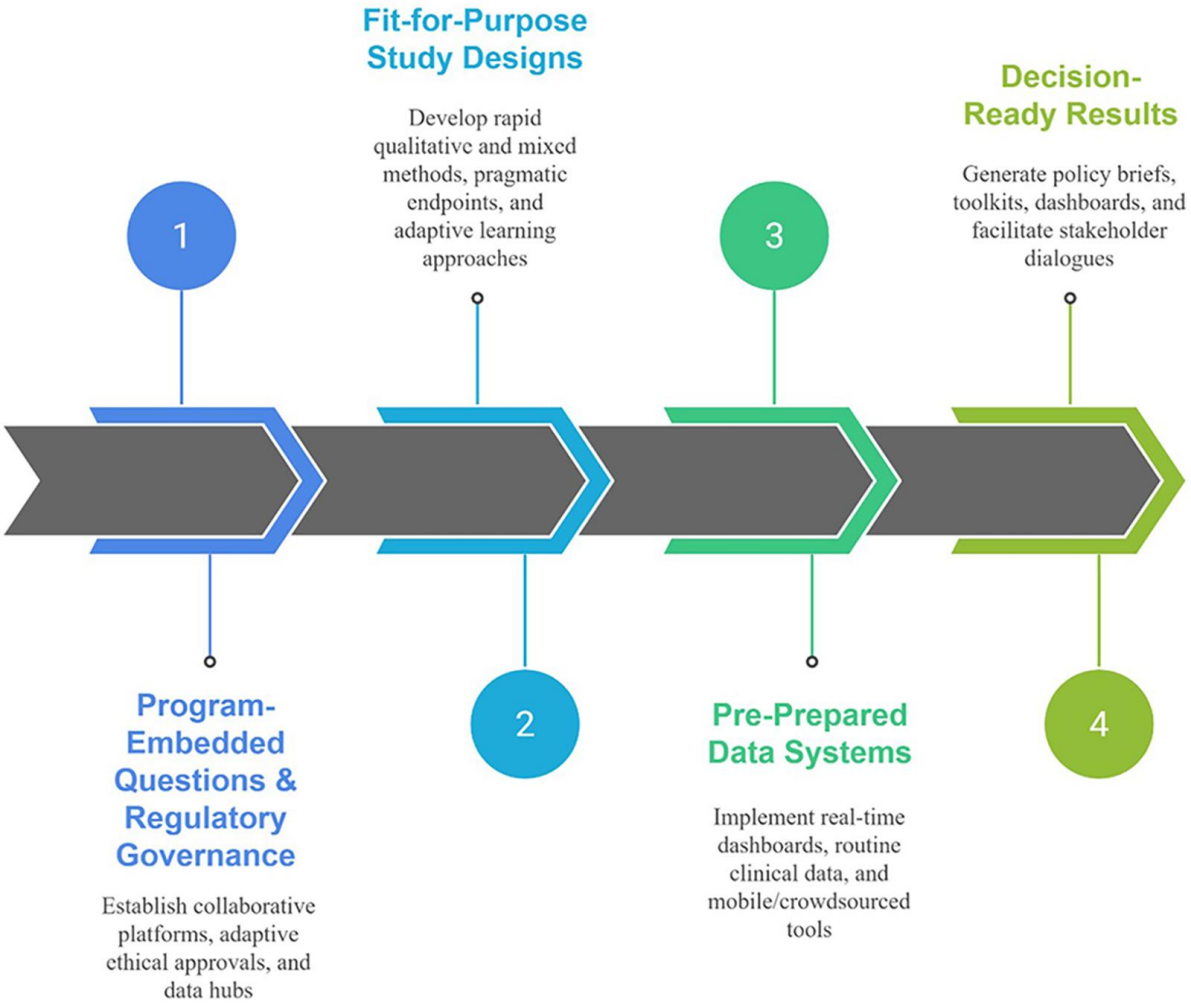
Four-step cascade for operationalizing rapid implementation science within HIV and public health programs.

We propose a coherent four-step cascade through which RIS can be operationalized for HIV and other public health programs. Figure 1. The cascade comprises program-embedded question formulation and regulatory governance, fit-for-purpose study designs, Pre-prepared data systems with fast data collection techniques, and decision-ready results that inform program adaptation and scale. Each level of the cascade is supported by enabling structures and systems, including collaborative platforms, institutional governance mechanisms to minimize delays, pre-curated data systems, and innovative data collection methods. Table 1. While there is no “silver bullet,” this cascade, supported by enabling structures, can substantially enhance the speed and responsiveness of implementation research.

**Table 1:** Description, advantages, and pitfalls of enabling structures and systems for rapid implementation science in HIV and other public health programs.

Approach	Description	Advantages	Pitfalls
Embedded and collaborative stakeholder platforms	Establishing integrated researcher–implementer partnerships either through embedded roles or co-designed stakeholder forums to develop, implement, and iteratively refine research aligned with program priorities. This may include placing researchers within service delivery systems or creating structured platforms for reflection and co-development.	Promotes relevance and ownership, enables timely, context-specific inquiry, and strengthens relationships between researchers and implementers. Facilitates rapid data generation and responsiveness to program dynamics; leverages practitioner insights.	Requires sustained coordination and incentive alignment, which can be time- and resource-intensive to maintain. The potential for role confusion or conflicts of interest necessitates institutional flexibility and integration.
Institutional governance mechanisms that minimise delays	Establishing institutional processes for fast-track ethics and regulatory review, enabling real-time research in response to evolving public health needs.	Accelerate research initiation to increase alignment with policy windows and urgent implementation needs.	Risk of compromised rigour if review standards are overly relaxed; may require national-level reform.
Pre-curated data systems	Using existing, structured routine data for rapid analysis and learning through dashboards, EHRs, or curated databases.	Enables real-time monitoring and evaluation; reduces data collection delays.	Requires an upfront investment in data systems and interoperability to address data quality and completeness issues.
Innovative fast data collection techniques	Rapid, participatory methods like crowdsourcing or SMS-based surveys to gather implementation insights quickly.	Low cost, highly scalable; enables diverse input and fast turnaround; adaptable to routine program settings.	Limited depth or representativeness depends on engagement, quality and facilitation.
Decision-ready dissemination and feedback structures	Packaging and sharing rapid findings through policy briefs, dashboards, toolkits, stakeholder forums, and community-facing formats, with mechanisms for feedback from policymakers, implementers, providers, and end users.	Supports timely translation of evidence into program adaptation and community uptake. Promotes shared interpretation of findings and strengthens the likelihood that evidence is acted upon	Dissemination may remain top-down if not paired with dialogue and feedback. Community-facing dissemination may be limited by low trust or weak engagement structures. Requires careful tailoring of messages for different audiences.

### Step 1: program-embedded research questions and regulatory governance

To operationalize RIS that supports programmatic decision-making, it is essential to establish structures that align the scientific cycle with programmatic timelines and ensure that research questions reflect program priorities. This raises the issue of who is best positioned to define research questions within ongoing programs. Addressing this requires deliberate engagement of key stakeholders. Collaborative platforms co-led by implementers, scientists, and community partners should be established to embed research questions within routine program activities and to inform strategy in real time. These collaborative platforms can support iterative learning and a space for development of shared goals.

Continuous collaboration among stakeholders (academics, implementers and policymakers) increases the use of research in policy and programming (8). All stakeholders must be engaged from the beginning. Meaningful stakeholder engagement should be systematically planned, implemented, and evaluated rather than being *ad hoc* or superficial (9). These collaborative platforms can leverage existing stakeholder engagement structures, like community advisory boards. Research questions should be co-developed within these platforms to ensure stakeholder input and relevance. However, meaningful stakeholder engagement must be balanced with the need for rapid question formulation. Rapid engagement does not necessarily require lengthy or repeated full-team deliberations to generate a research question. Instead, programs can use the pre-existing stakeholder platforms through brief structured consultations and asynchronous feedback mechanisms to quickly identify urgent uncertainties in the program. This allows frontline implementers, policymakers, community representatives, and researchers to provide early input on what questions matter most, while smaller technical teams can subsequently refine these priorities into scientifically answerable research questions. Frontline implementers, though often aware of how innovations influence practice, may lack the capacity to formulate research questions. Therefore, their insights when generating research questions are important. In this way, stakeholder engagement is treated as rapid but broad enough to ensure relevance.

Glasgow et al. exemplify this approach through an iterative, stakeholder-driven process. In their study, an interdisciplinary implementation team, including investigators, program coordinators, frontline staff, and analysts, jointly identified and prioritized key research questions across five health service projects. Using the Reach, Effectiveness, Adoption, Implementation, and Maintenance (REAIM) framework, regular meetings fostered collaborative reflection and allowed implementers to continuously evaluate the projects along the REAIM domains, prioritise focus areas, and establish mid-course goals to improve outcomes (10). Similarly, Quinn and colleagues describe a co-creative process in which academics, government officials, and implementation partners collaboratively developed standardised indicators and data-collection tools for clean-cooking programs across eleven countries. This process involved iterative consultations and co-design of templates tailored to local contexts. The insights and experiences of frontline implementers, policy advisors, and development partners shaped these tools from the outset (11).

Integrating governance mechanisms into collaborative platforms within public health programs is also essential for RIS. A compelling example comes from Zambia, where a Ministry of Health-led data hub, established with technical support from PEPFAR, functions as a real-time analytics platform (12). The hub rapidly interrogates program data to inform strategic planning, assess gaps, and guide implementation decisions. Another major barrier to RIS is the prolonged ethical and regulatory approval processes, which often fail to match the urgency of real-time public health response (13). As Dzinamarira et al. showed across various emergencies in Africa, streamlined ethical procedures, including expedited research ethics committee processes and adaptive review mechanisms, were crucial in enabling the timely deployment of digital health tools (14). Expedited governance mechanisms must not be interpreted as diminished ethical oversight. On the contrary, rapid implementation research requires proportionate, adaptive ethics review processes that preserve participant protection, community accountability, and informed consent, particularly in donor-funded and resource-constrained settings where power asymmetries may be pronounced.

Despite the success of these kinds of models, establishing and operationalizing collaborative platforms can be challenging. To sustain meaningful collaboration, tangible incentives are required to align the interests of programming and research communities. This includes funding opportunities, professional recognition, and shared resources. By incentivizing engagement, fostering a cooperative environment, and demonstrating the economic dividend/cost savings to policy makers, these platforms can significantly improve the agility and impact of the HIV response and broaden public health initiatives.

### Step 2: fit-for-purpose rapid study designs and endpoints

A growing set of qualitative and quantitative study designs supports the goals of RIS, particularly those that enable iterative testing, flexible data collection, adaptation during implementation, early assessment of proximal outcomes, and the use of pragmatic endpoints to generate timely, decision-relevant evidence for public health programs. While accelerated timelines may necessitate methodological trade-offs to enable timely results, these trade-offs must be made explicit, documented, and justified, with transparency regarding limitations, bias, and uncertainty, while maintaining analytic discipline, reproducibility, and scientific integrity.

#### Qualitative study designs

A notable body of work has demonstrated the utility of qualitative Rapid Assessment Procedures (RAP) for program-responsive inquiry (15). RAP has been used to develop an HIV/AIDS prevention program tailored to how Latinas perceive virus transmission (16). It has also supported formative evaluations, including UNICEF programs aimed at mid-course improvements in program effectiveness, and assessments of the impact of primary health care interventions on nutrition and family health (17). More recently, RAP has been applied to the evaluation of implementation efforts (18, 19). Ackerman and colleagues used “rapid ethnography” to investigate the adoption of patient portals in California safety-net health systems through interviews, observations, and review of site materials (20).

The Rapid Research Evaluation and Appraisal Lab (RREAL) at University College London has advanced the application of rapid qualitative methods in time-sensitive global health contexts, contributing to methodological innovations, such as hybrid models blending ethnographic immersion with structured templates to accelerate data synthesis (21, 22). RREAL has operationalized structured, team-based approaches, like the use of RREAL Sheets and concurrent data collection and analysis cycles, to inform decision-making during ongoing health interventions (23). Their work during the COVID-19 pandemic demonstrated how rapid appraisals could produce actionable insights for healthcare system responses within weeks, not months, while maintaining rigour and transparency (24).

Finley and colleagues have taken a step further to incorporate ethnography into the continuous or timely evaluation of programs by using a method called periodic reflections (25). This method utilises structured qualitative interviews conducted monthly or bimonthly with the implementation core team over a 30-minute phone call to assess progress and capture events as they occur, with opportunities for adjustment. Unlike formal interview guides, the periodic reflection method uses a brief, guided discussion template that encourages team members to reflect on ongoing activities, recent challenges, and adaptations to the intervention plan.

Beyond purely qualitative approaches, mixed-method designs that integrate implementation science frameworks have also been developed that could support RIS. One such approach is the REAIM QuEST (Reach, Effectiveness, Adoption, Implementation, and Maintenance Qualitative Evaluation for Systematic Translation) tool, which has been used to integrate qualitative inquiry into the REAIM framework domains (26). RE-AIM QuEST is designed for formative use in real time during implementation, providing a mechanism to rapidly identify and respond to emerging barriers, tailor implementation strategies, and optimise outcomes as the program unfolds. The AIM study (27, 28), a multi-site randomised trial on adherence and blood pressure control demonstrated the value of this approach. Weekly monitoring of enrollment metrics flagged underperformance at one site; however, quantitative data alone did not explain the reason. Qualitative feedback from implementers revealed staffing shortages and uneven pharmacist workloads as key barriers, insights that guided timely program adjustments.

Similarly, the Rapid Assessment Procedure-Informed Clinical Ethnography (RAPICE) method is a valuable tool in pragmatic clinical trials, focusing on real-world applicability and quick evidence generation (29). It combines ethnographic immersion with rapid assessment techniques, utilising multidisciplinary teams trained in qualitative methods and various data collection tools such as participant observation and semi-structured interviews. RAPICE is iterative, allowing continuous refinement of research questions and strategies. When appropriately adopted, these methodological approaches can support real-time adaptation, which is crucial in Low- and middle-income countries (LMIC) settings where implementation contexts are rapidly evolving.

#### Quantitative study designs

Quantitative study designs can provide practical, timely approaches to facilitate RIS. Smith and colleagues described three methods that can be adapted for RIS: the analysis of short-term outcomes, the extrapolation of early or existing evidence to estimate longer-term effects, and real-time monitoring of process and outcome measures to enable continuous feedback and program adjustment (5).

First, through evaluating short-term outcomes, researchers can measure observable changes within a limited period. It requires finding proxy interim outcomes that balance credibility and validity as indicators of downstream events (5). For example, proxy outcomes, such as medication pickup, can serve as indicators of adherence, and service uptake could be an early indicator of behavioural change. These outcomes can be assessed to generate timely insights before long-term effects manifest. Relevant RIS within programs is impossible if you choose a clinical endpoint that takes two years to occur. Traditional scientific designs, such as A/B testing, can effectively facilitate the assessment of useful short-term outcomes across multiple strategies to select the one likely to maximise outputs early on. A/B testing has been used to identify and evaluate strategies to improve service quality and the performance of clinical trial recruitment websites (30). It can be embedded within program workflows, like rapidly testing whether daily SMS reminders or weekly calls can improve PrEP delivery programs within a month to generate evidence that informs prompt program decisions. Stepped wedge designs can also be effectively adapted for this purpose. Through implementing interventions across sites in swift, successive phases, teams can begin to evaluate impacts early while continuing the rollout in other locations. Minimising the interval between rollout phases can accelerate feedback loops, allowing program teams to identify early indicators of effectiveness or potential barriers and make informed adjustments for subsequent sites. These adaptations can transform the stepped wedge design into a dynamic, embedded approach that supports rapid, iterative improvements alongside program scale-up.

Secondly, extrapolating short-term outcomes through mathematical modelling can accelerate decision-making by estimating future impacts before long-term outcomes are observable (31). Simulation models synthesize available evidence to anticipate downstream consequences of an intervention, allowing implementers to foresee potential benefits or unintended effects early. The WHO guideline developers note that modelling studies can extend trial results to project long-term outcomes and impacts in different settings (32). Mathematical modelling can thus support rapid implementation by guiding scale-up and adaptation without waiting years for definitive endpoint data.

Thirdly, continuous monitoring through sequential time series enables real-time tracking of program performance, identifying issues early and adjusting implementation accordingly. This approach can utilise existing Quality Improvement (QI) infrastructure (33). In QI science, data are routinely analysed over time using statistical process control charts to determine if changes are leading to improvement. This rapid feedback loop allows implementers to learn and adjust quickly. Using Plan-Do-Study-Act cycles, teams make data-driven modifications, ensuring that ineffective ideas are adapted or abandoned early, thereby preventing wasted resources (33). Importantly, many health systems already maintain QI platforms with built-in data reporting, audit-and-feedback loops, and provider-engagement mechanisms. Leveraging existing real-time data systems enables rapid improvement without additional cost or new infrastructure, effectively integrating research and evaluation into routine practice.

### Step 3: pre-prepared data systems and fast data collection techniques

Rigid, lengthy data-collection processes often delay results until after programs have been implemented. Data collection should be streamlined to provide timely insights to guide program adjustments. Large volumes of routine clinical data offer valuable opportunities to meet this. When integrated into real-time dashboards, it can support rapid decision-making. Existing database, including Data for Accountability, Transparency, and Impact Monitoring (DATIM) (34) and the International Epidemiology Database to Evaluate AIDS (IeDEA) (35) can be leveraged for this purpose.

The IeDEA–World Health Organization (WHO) collaboration exemplifies how routine clinical data can drive real-time decision-making in global health programs (36). IeDEA actively collects and harmonises patient-level data from over 1.7 million individuals across 46 countries, on key HIV care outcomes such as ART initiation, retention, and viral suppression. Analyses of this data revealed critical trends, like a steady shift toward earlier enrolment in HIV care over time, and a declining proportion of people retained on ART within the first five years. These insights would have been missed without longitudinal, harmonised data systems. This collaboration has allowed WHO to rapidly assess the implementation of new HIV guidelines and identify service delivery gaps, with many analyses completed and delivered within six months of data collection. The resulting evidence has directly informed WHO's global HIV progress reports and supported guideline updates.

Geng et al. utilized routinely collected clinic-based data from electronic records and pharmacy systems in Uganda to evaluate program performance in real-time. In this study at the ISS Clinic in Southwestern Uganda, they identified sharp declines in antiretroviral treatment initiation following changes in donor funding, tracing these patterns down to individual stakeholders by drawing exclusively on existing operational data (37). This demonstrates the importance of designing data systems that serve both clinical and evaluative functions, enabling programs to ask and answer urgent implementation questions without delay.

Innovative, fast, and low-cost data collection methods, like mobile phones and crowdsourcing, can also facilitate rapid research within programs. Crowdsourcing is an open invitation to public participation in problem-solving, which has gained traction in health research. It has been applied to a range of activities, including data collection, analysis, surveillance, and surveying (38). It can be leveraged for fast data collection to facilitate rapid implementation science. Davis et al. facilitated a 10-minute exercise at a conference, where diverse attendees generated over 30 high-quality messages for sexually transmitted infection testing, which were quickly judged and shared online (39). Such methods could be integrated into stakeholder meetings or community forums.

Odeny et al. also provide a compelling example of how low-cost mobile technology can accelerate data collection in programmatic settings (40). They utilised SMS-based surveys to map health worker networks in Kenya, achieving a 97% response rate in just three days at a cost of less than $10 (40). The entire process was built on open-source platforms requiring no prior programming expertise. Alternative data collection strategies, particularly those utilising widely available mobile tools, can facilitate rapid, scalable insights in real-world settings, making them highly suitable for embedded or rapid-implementation research.

### Step 4: decision-ready results for program adaptation and scale

To ensure rapid evidence informs program decisions and adoption, results must be packaged in accessible and timely formats through both technical and community-facing pathways. Policy briefs, dashboards, toolkits, and stakeholder forums are key dissemination tools for technical teams to translate findings into programs. Community-facing dissemination should use plain-language formats and trusted local messengers, with rapid feedback mechanisms so that evidence can be understood, challenged, and used by clients and communities.

Policy briefs are concise, targeted summaries of research findings and recommendations tailored for non-technical audiences. They have become a widely used mechanism for distilling complex evidence into key findings and clear recommendations that policymakers can quickly absorb (41). The WHO routinely releases policy briefs alongside new HIV guidelines or emerging evidence to support countries in integrating recommendations into national programs. For example, a policy brief was used to introduce the WHO framework for the “triple elimination” of mother-to-child transmission of HIV, syphilis, and hepatitis B, succinctly outlining the framework's concepts and policy steps for country adoption (42). However, evidence suggests that policy briefs are most effective when paired with opportunities for dialogue; dissemination alone has limited impact unless accompanied by discussion and contextualization (43). As such, policy briefs should be followed by stakeholder meetings or policy dialogues that allow their content to be questioned, interpreted, and adapted to local realities, reinforcing the role of the collaborative platforms described earlier in the cascade.

Rather than simply circulating reports or academic publications, these platforms bring together researchers, policymakers, program implementers, community representatives, and other stakeholders to jointly interpret evidence and plan action (9). Many countries and international agencies have institutionalized such engagements through national HIV research symposiums, evidence-to-policy roundtables, and global conferences or implementation workshops focused on translating science into practice. A scoping review found that stakeholder feedback meetings increase the perceived relevance and credibility of research among decision-makers and improve the likelihood that findings are used in policy or practice (43). This collaborative approach supports adaptive programming by enabling real-time program modifications informed by those responsible for implementation and those affected by it.

Besides policy briefs, results can also be packaged as implementation toolkits (44). These toolkits typically include step-by-step implementation guidance, training materials, checklists, case studies, and adaptation tips grounded in evidence and best practice. They offer a ready-to-use blueprint that allows program planners and service providers to adopt new interventions more rapidly while maintaining fidelity to core principles (44). WHO and its partners have produced several influential HIV implementation toolkits, including the HIV self-testing implementation toolkit launched in 2023–2024, which provides technical guidance for introducing and scaling self-testing for HIV (and, where relevant, hepatitis C and syphilis) (45). Similarly, the WHO PrEP implementation tool supports the rollout of pre-exposure prophylaxis through modules on service delivery models, demand creation, and monitoring.

Interactive data dashboards represent another powerful dissemination approach, particularly in an era of data-driven programming. These web-based platforms visualize epidemiological and program data in real or near-real time, enabling users to explore trends, disaggregate data by population or geography, and identify gaps requiring action. Global HIV initiatives have increasingly adopted dashboards to track progress toward targets and inform program adjustments. For example, WHO, in coordination with UNAIDS, introduced an HIV Testing Services dashboard that compiles global and country-level data on testing uptake, yield, prevalence, and the use of newer approaches such as self-testing (46). Presented through accessible charts and tables, the dashboard supports practical decision-making. In the United States, the America's HIV Epidemic Analysis Dashboard (AHEAD) tracks jurisdiction-level progress toward Ending the HIV Epidemic targets, and the IeDEA Treat All Dashboard in sub-Saharan Africa was explicitly designed to make treatment data accessible to decision-makers at facility, district, and national levels (36). The strength of interactive dashboards lies in their immediacy and usability, transforming complex datasets into intuitive visualizations that enable policymakers to assess program performance and respond accordingly quickly.

Community-facing dissemination is also critical if decision-ready results are to support client understanding, trust, and uptake. Although we found limited direct examples in the literature of rapid dissemination of implementation findings to communities, HIV implementation studies suggest that strategies are more effective when embedded within existing community networks and delivered through trusted messengers. For example, a peer-led HIV prevention intervention among youth experiencing homelessness demonstrated that network position and messenger selection influenced the speed of behaviour change, with earlier effects observed when peer leaders were selected using network-informed approaches (47). This study does not directly evaluate dissemination of research findings but supports the principle that community-facing dissemination should use existing trusted structures, peers, and community actors with social legitimacy to translate evidence into forms that are understandable, credible, and actionable for end users.

### Recommendations and future directions

To institutionalise RIS within health programs, adopting innovative rapid methods and adapting traditional research designs to deliver timely insights is essential. Study designs should explicitly balance speed and rigour by using relevant, interim, and pragmatic outcomes that support early strategic adjustment and be supported by enabling structures that facilitate collaboration among implementers, policymakers, and researchers within service delivery systems. Investment in pre-prepared, analysis-ready data systems integrated with routine care is critical to enable real-time insights, leveraging existing digital tools and QI infrastructure for continuous monitoring and feedback. Emerging artificial intelligence (AI)–enabled analytic tools further expand opportunities to democratize access to advanced analytics by automating data cleaning, visualization, and modelling, enabling frontline implementers and managers to generate timely program intelligence and iterate interventions in real time.

Future efforts should advance hybrid models that combine rapid methods with embedded learning cycles, strengthen locally led research capacity, and explicitly assess trade-offs between timeliness, generalizability, and scientific quality. In doing so, RIS can evolve into a core institutional practice that is not only scientifically robust but also financially and politically responsive. As donor resources become increasingly constrained, demonstrating how RIS improves efficiency, reduces waste, and translates investments into measurable outcomes will be essential, and these contributions should be systematically assessed and documented. This could include indicators such as time from question formulation to decision-ready evidence, cost per evaluation, use of routine or pre-prepared data systems, resources reallocated based on rapid evidence, and measurable changes in program performance.

## Conclusion

Rapid Implementation Science can be systematically operationalized within HIV and other public health programs to align scientific processes with programmatic decision cycles. By making explicit and justified trade-offs between speed, rigor, and generalizability, RIS provides a structured approach for producing evidence that remains scientifically credible while being timely enough to influence real-world program decisions. This necessitates embedding implementation research within programs through the creation of collaborative platforms that support program-embedded formulation of research questions, the use of fit-for-purpose study designs with pragmatic endpoints, investment in pre-prepared data systems coupled with fast data collection methods, and the generation of decision-ready results through both technical and community-facing pathways that directly inform program adaptation and scale. Together, these approaches embed rapid scientific inquiry within dynamic program timelines, enabling evidence to function as real-time input for program management rather than a retrospective evaluation.

## Data Availability

The original contributions presented in the study are included in the article/Supplementary Material, further inquiries can be directed to the corresponding author.
